# Correction: Iannuzzi et al. Fyn Tyrosine Kinase Elicits Amyloid Precursor Protein Tyr682 Phosphorylation in Neurons from Alzheimer’s Disease Patients. *Cells* 2020, *9*, 1807

**DOI:** 10.3390/cells13121026

**Published:** 2024-06-13

**Authors:** Filomena Iannuzzi, Rossana Sirabella, Nadia Canu, Thorsten J. Maier, Lucio Annunziato, Carmela Matrone

**Affiliations:** 1 Department of Biomedicine, Aarhus University, Aarhus C, 8000 Aarhus, Denmark; filomena.iannuzzi@biomed.au.dk; 2 Division of Pharmacology, Department of Neuroscience, School of Medicine, University of Naples Federico II, 80131 Naples, Italy; sirabell@unina.it; 3 Department of System Medicine, University of Rome “Tor Vergata”, 00133 Rome, Italy; nadia.canu@uniroma2.it; 4 Institute of Biochemistry and Cell Biology, CNR, 00015 Monterotondo, Rome, Italy; 5 Paul-Ehrlich-Institut, (Federal Institute for Vaccines and Biomedicines), 63225 Langen, Germany; thorstenjuergen.maier@pei.de; 6 SDN Research Institute Diagnostics and Nuclear (IRCCS SDN), Gianturco, 80131 Naples, Italy

**Error in Figure**

In the original publication [1], there was a mistake in *Figure 5* as published. Specifically, Western blots labeled with β-actin in the middle and right panels of 5 B and APP in the middle panel of 5 B were placed incorrectly during assembly. In addition, the Western blot labeled with pTyr (Figure 5A, left) was mistakenly flipped horizontally. The corrected panels and the corresponding optical density analyses are shown below. The authors apologize for the inconvenience and state that the scientific conclusions are unaffected. This correction was approved by the Academic Editor. The original publication has also been updated.

## Figures and Tables

**Figure 5:**
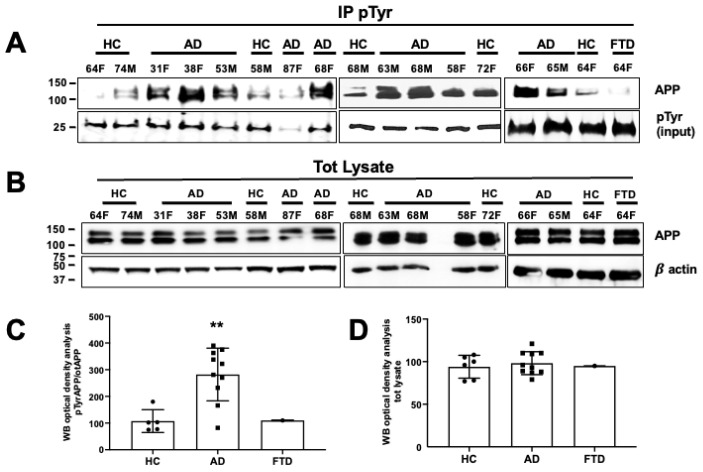
The amyloid precursor protein was phosphorylated at the Tyr residue in neurons from AD patients. (**A**) After 5 weeks in differentiating media, neurons from healthy controls (HCs) and AD patients and one FTD patient were processed for pTyr IP using mouse anti-pTyr magnetic beads- conjugated antibody (4G10) and analyzed by WB using rabbit anti-APP antibody (Y188). Membranes were blotted with anti-mouse IgG, and the pTyr band (input) migrating at 25 kDa was used as a loading control. (**B**) WB analysis of basal APP levels using the rabbit anti-APP antibody (Y188). β-actin was used as a loading control. (**C**,**D**) Densitometric analysis of pAPP-Tyr levels in AD patients vs. HCs (**C**) and total APP levels expressed as a percentage of HCs (**D**). Mean optical density values were calculated as the ratio of pAPP-Tyr levels relative to basal APP levels (after normalization to β-actin) from each sample (experiments from each sample were repeated three times, *n* = 3). Statistically significant differences were calculated using Student’s *t*-test. ** *p* < 0.01 vs. HCs.
