# Supplementary material for: Fyn Tyrosine Kinase Elicits Amyloid Precursor Protein Tyr682 Phosphorylation in Neurons from Alzheimer’s Disease Patients
Source: Cells. 2020 Jul 30;9(8):1807. doi: 10.3390/cells9081807 (PMC7463977; doi:10.3390/cells9081807)
Supplement: Supplementary file 1 [file cells-09-01807-s001.pdf]

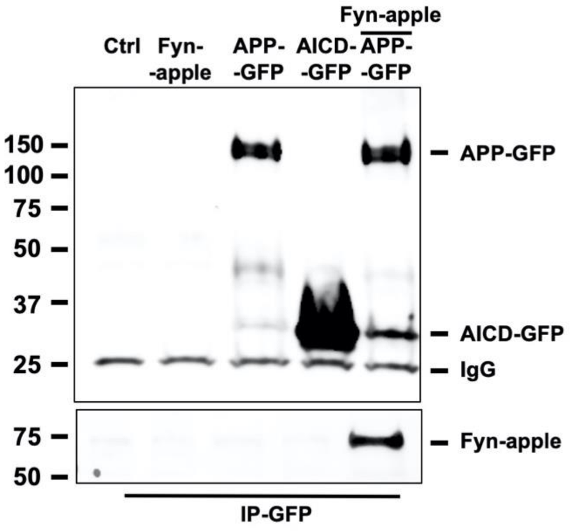

Supplementary Figure S1: APP and Fyn interact in APP-GFP+Fyn-apple overexpressing neurons. Ctrl, Fyn-apple APP-GFP, AICD-GFP, APP-GFP+Fyn-apple neurons were immunoprecipitated with anti GFP antibody and analyzed with anti-APP or anti-Fyn antibody. As expected, a band corresponding to APP-GFP, migrating approximately at 150KDa, was evident only in the APP-GFP and APP-GFP+Fyn-apple lanes, demonstrating the efficiency of the transfection procedure. AICD-GFP (AICD) was used as a molecular weight internal control to identify AICD-GFP peptide that was produced in APP-GFP+Fyn-apple neurons. Of interest, the appearance of Fyn-apple in the APP-GFP+Fyn-apple lysates indicates that Fyn interacts with APP when both APP and Fyn are overexpressed. The experiment is representative of three independent experiments.

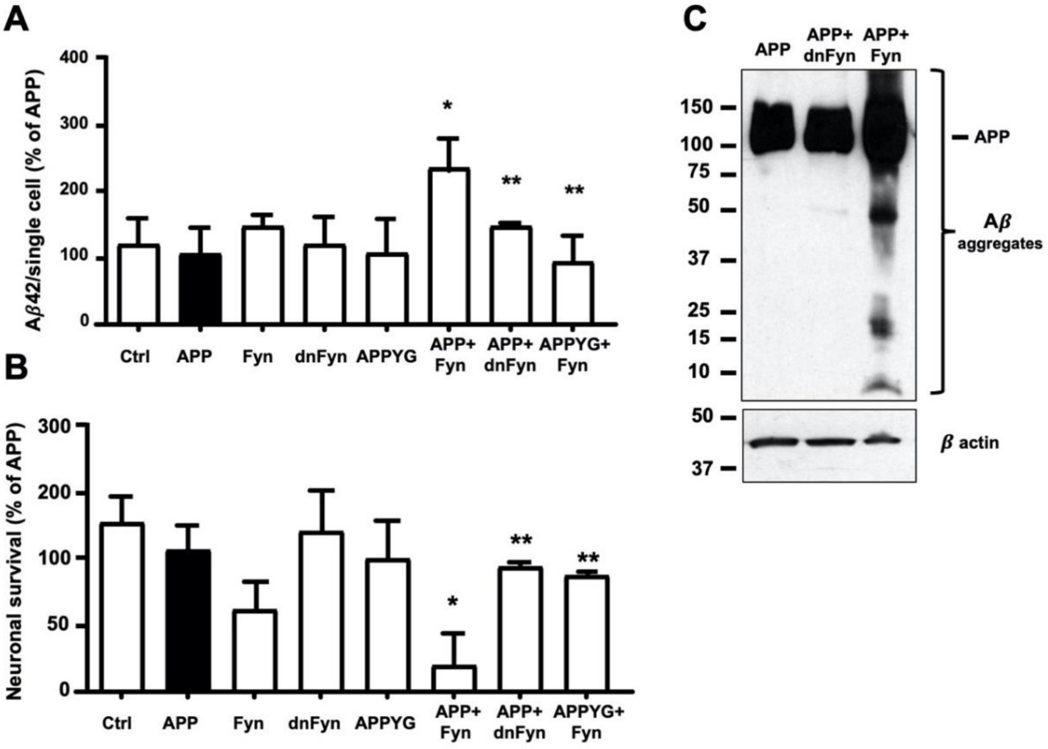

Supplementary Figure S2: Fyn promoted APP phosphorylation at Tyr682 in human neurons. (**A**) ELISA analysis of Aβ42 from media of Ctrl, APP, Fyn, and APP+Fyn transfected neurons. Aβ42 levels

were normalized to the number of alive cells (DAPI stained nuclei) that were present on each slide after 48 h of transfection and are expressed as ng of Aβ42 that were released from each cell in 0.5 ml of media. Data are reported in as % of APP transfected neurons. Each experiment was performed three times in triplicate ($n = 3$). *$p \leq 0.05$, vs. APP (black bar). Statistically significant differences were calculated using one-way ANOVA followed by Dunnett's post hoc test. (**B**) Transfected neurons and controls were stained with DAPI, and the number of alive neurons was counted under an immunofluorescence microscope. Each experiment was performed three times in triplicate ($n = 3$). The data are expressed as a percentage of APP. *$p < 0.05$, vs. APP (black bar). Statistically significant differences were calculated using one-way ANOVA followed by Dunnett's post hoc test. (**C**) WB analysis with anti Aβ antibody (4G8 clone, Millipore) from total lysate of APP, APP+dnFyn and APP+Fyn transfected neurons. The experiment reported in C is representative of three independent experiments.
